# Evaluation of the Effect of Body Position on Intraocular Pressure Measured with Rebound Tonometer

**DOI:** 10.4274/tjo.galenos.2018.90359

**Published:** 2019-02-28

**Authors:** Hüseyin Mayalı, Beyza Tekin, Özcan Rasim Kayıkçıoğlu, Emin Kurt, Süleyman Sami İlker

**Affiliations:** 1 Manisa Celal Bayar University Faculty of Medicine, Department of Ophthalmology, Manisa, Turkey

**Keywords:** Intraocular pressure, body position, tonometer

## Abstract

**Objectives:**

It is important to determine variables that influence intraocular pressure (IOP) measurement. This study aimed to evaluate the effect of body position on IOP.

**Materials and Methods:**

The study included 52 right eyes of 52 patients who presented to the ophthalmology department of our hospital and had no ocular disease except refractive errors. IOP was measured with an Icare PRO tonometer while patients were in sitting, standing, and supine positions, with intervals of 10 minutes between the positions. Correlations between the results were evaluated using Spearman’s correlation analysis and Wilcoxon tests.

**Results:**

Thirty-six of the 52 patients were female, 16 were male. Mean age was 31.65±6.30 (23-47) years. Mean IOP values in the sitting, standing, and lying positions were 17.76±3.41 (12.70-25.60) mmHg, 17.10±3.27 (11.50-25.20) mmHg, and 18.46±4.67 (10.50-29.40) mmHg, respectively. There were no statistically significant differences between measurements taken in the different positions (p=0.112, p=0.472, p=0.071). We observed that there was no relationship between age and body position (p>0.45, p>0.79, p>0.77) or between gender and position (p>0.59, p>0.69, p>0.54).

**Conclusion:**

Gender and age had no effect on IOP measured in different body positions. There were also no significant differences between IOP values measured in the different positions. Therefore, we believe the portable Icare PRO tonometer can be used for patients who are confined to bed and will provide IOP measurements that are concordant with values obtained while sitting.

## Introduction

Glaucoma is one of the causes of substantial visual loss due to optic nerve injury. High intraocular pressure (IOP) is the most important risk factor. However, IOP is dynamic and can be affected by many factors. Pronounced changes in IOP in horizontal body position have been demonstrated in previous studies, and people spend one third of their lives lying down.^1,2,3,4,5,6^

Positional changes in IOP may be important in the development and course of glaucoma. Previous studies have also demonstrated wide variation in the difference between IOP values obtained in supine position and sitting position. This difference varies between 0.3 mmHg and 5.6 mmHg in studies evaluating healthy individuals and patients with glaucoma.^7,8,9,10,11,12,13,14,15,16,17^

The physiology of postural changes in IOP is not fully understood. Understanding IOP changes related to body position may be important in order to understand the development and course of glaucoma, determine variations in IOP measurements obtained in clinical follow-up, and provide more standardized follow-up. Furthermore, if the nature of these effects is better understood, glaucoma patients can be advised about what situations they should avoid in their daily lives or which may benefit them. 

In this study, we measured IOP in healthy individuals in sitting, standing, and supine position and evaluated differences in IOP between these positions.

## Materials and Methods

The study was carried out in accordance with the principles of clinical research set forth in the Declaration of Helsinki and was approved by the Ethics Committee of Manisa Celal Bayar University Faculty of Medicine. Fifty-two right eyes of 52 individuals who presented to the ophthalmology department of the Manisa Celal Bayar University Hafsa Sultan Faculty of Medicine with refractive errors not exceeding -4.00 and +2.00 were included in the study. Patients using systemic or topical medication and those with ocular surface disease, uveitis, glaucoma, retinal detachment, ocular infection, and strabismus were not included. A detailed ophthalmologic examination was performed before the study to identify individuals who met these criteria. In addition, the nature of the study was explained verbally to each participant and informed consent was obtained from all participants before the study. Participants were instructed to sleep normally the night before and to abstain from excessive caffeine intake on the day of the study.

The Icare PRO rebound tonometer (Icare; Tiolat Oy, Helsinki, Finland) was used in this study. Subjects were seated for 10 minutes, after which 6 serial IOP measurements were taken in quick succession from their right eye with the Icare PRO tonometer while they remained in sitting position. The average of these 6 measurements was used. The subjects were then asked to stand for 10 minutes, after which 6 serial IOP measurements and their average value were obtained as before. Finally, the patients laid in supine position on the clinic stretcher with no pillow for 10 minutes, after which the same IOP measurement procedure was repeated. Subjects were encouraged to relax in order to avoid actions that would increase pressure on the eyelids or globe during measurements. Based on the color-coded measurement reliability system in the Icare PRO tonometer, we only used average values that were green, indicating low variability and high reliability. The Icare PRO includes an automatic system that compares 6 manual measurements, evaluating variation between them and calculating an average. Green indicates lowest variability and highest reliability, yellow indicates moderate variability and reliability, and red indicates high variability and low reliability.^2,3,4,5,6,7,8,9,10,11,12,13,14,15,16,17,18^

### Statistical Analysis

Using the SPSS program, normality of the sample set was evaluated and the non-parametric Spearman’s correlation test and Wilcoxon test were used to statistically evaluate relationships between the participants’ age and sex, respectively, and the different body positions. P values <0.05 were considered statistically significant.

## Results

Of the 52 participants, 36 were female and 16 were male; their mean age was 31.65±6.30 (23-47) years. Table 1 shows that the sample set was not normally distributed. Table 22 shows the mean IOP values obtained in sitting, standing, and supine positions and statistical comparisons between these values using Wilcoxon test.

A p value >0.05 in this test of normality indicated that the group was not distributed normally. Therefore, non-parametric tests were used in all further statistical analyses.

There were no statistically significant differences in IOP values obtained in sitting when compared with values obtained in standing and supine position (p=0.112, p=0.472). There was also no significant difference in the comparison of standing and supine position (p=0.071).

The relationship between the participants’ age distribution and IOP in different body positions was examined using Spearman’s correlation test (Table 3) and the relationship between sex and IOP in different body positions was examined using the Wilcoxon test (Table 4).

No relationship was observed between age and IOP measured in sitting, standing, and supine positions (p=0.45, p=0.79, p=0.77). There was a positive correlation between age and IOP in sitting and standing position, while a negative correlation was observed in supine position. Relationships between sex and IOP measured in sitting, standing, and supine positions were not statistically significant according to the results of the Wilcoxon test (p=0.59, p=0.69, p=0.54).

## Discussion

Changes in IOP occurring with changes in body position have been evaluated in numerous studies over the years, with emphasis that this issue may be important for patients with glaucoma. Previous studies have shown that IOP differs significantly between sitting and supine position and that IOP is higher in supine position compared to sitting position. Furthermore, these studies reported that the difference in IOP between sitting and supine position was more pronounced in glaucoma patients. The magnitude of this difference is 0.3-5.6 mmHg in healthy individuals and patients with glaucoma.^1,7,8,9,10,11,12,13,14,15,16,17^

The physiology of posture-induced changes in IOP has not been fully elucidated. However, in another study involving 24-hour observation, it was emphasized that IOP has a circadian rhythm. The authors reported a change of 4.5-20 mmHg between IOP values taken at night in supine position and in the day in sitting position. This indicates that IOP measurements in glaucoma patients should be performed at similar times of day and also suggests that IOP spikes that may accelerate glaucoma progression could go undetected and unnoticed by clinicians.^2,19^

Axial length, which is believed to be a factor in the physiology of IOP change, has also been evaluated in some studies. The increase in intraocular pressure when moving from sitting to supine position was found to be greater in patients with short axial length and smaller increases were observed in patients with myopic defocus greater than -4.00 diopters.^1,20,21^

In numerous studies, the increase in episcleral venous pressure (EVP) that occurs when lying down was proposed as an explanation of this change in IOP. However, these studies were unable to show an exact correlation between EVP increase and expected IOP increase or clearly demonstrate whether IOP increase was a result of the EVP increase or other factors.^22,23,24,25^

Different methods of evaluating IOP changes according to body position have been described in the literature. In healthy subjects, the increase in IOP between sitting and supine position was reported as 1.8 mmHg using Perkins applanation tonometer, 2.5-3.9 mmHg with pneumotonometer, 1.2 mmHg with Tono-Pen, and 4.1 mmHg with Goldman applanation tonometry.^1,26,27,28,29^ Mosaed et al.^30^ reported relatively small postural change in IOP in healthy young adults and elderly individuals with healthy eyes, while another study reported that postural IOP changes in these two populations were nonsignificant.^31^

Although our study did not yield any findings that support previous studies, we can say that changes in IOP are not related to body position. Because our study included only healthy young adults, the same results may not be obtained in elderly individuals or glaucoma patients. Our investigation focused on the relationship between IOP and body position, and we observed a difference of 0.7 mmHg between sitting and supine positions. A limitation of our study is that we did not control for systemic parameters such as blood pressure, heart rate, and central venous pressure.

Our results indicated that there was no statistically significant difference in IOP measured in sitting and supine position with the Icare PRO tonometer. Therefore, we believe the Icare PRO tonometer may be appropriate for IOP monitoring in glaucoma patients who are confined to bed. The other main finding of our study is that differences in IOP values when sitting, standing, and in supine position are independent of sex and age.

### Study Limitations

In our study, we used the Icare PRO tonometer to measure IOP in randomly selected healthy individuals in a specific order (sitting, then standing, then supine). This was important in terms of standardizing the measurement process between participants. However, the relatively small sample and inclusion of only healthy individuals were among the limitations of this study.

## Conclusion

A more comprehensive study is needed to understand how IOP changes with respect to position and time of day and to determine whether these factors affect glaucoma. These studies will help develop recommendations for glaucoma patients with comorbidities on how to optimize their living conditions.

## Figures and Tables

**Table 1:**
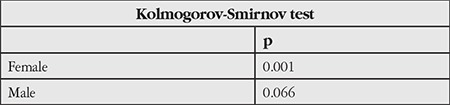
Normality test

**Table 2:**
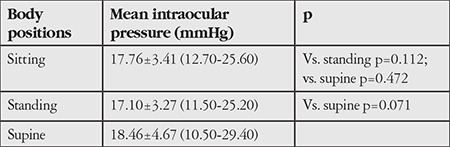
Statistical comparison of mean intraocular pressure values measured in different body positions

**Table 3:**
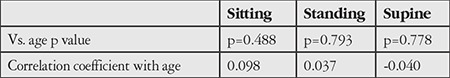
Relationship between age and intraocular pressures measured in sitting, standing, and supine positions

**Table 4:**
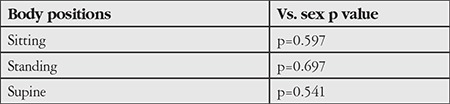
Relationship between sex and intraocular pressures measured in sitting, standing, and supine positions

## References

[1] Malihi M, Sit AJ (2012). Effect of Head and Body Position on Intraocular Pressure. Ophthalmology..

[2] Barkana Y, Gutfreund S (2014). Measurement of the difference in intraocular pressure between the sitting and lying body positions in healthy subjects: direct comparison of the Icare Pro with the Goldmann applanation tonometer, Pneumatonometer and Tonopen XL. Clin Exp Ophthalmol.

[3] Asrani S, Zeimer R, Wilensky J, Gieser D, Vitale S, Lindenmuth K (2000). Large diurnal fluctuations in intraocular pressure are an independent risk factor in patients with glaucoma. J Glaucoma..

[4] Krieglstein G, Langham ME (1975). Influence of body position on the intraocular pressure of normal and glaucomatous eyes. Ophthalmologica.

[5] Jain MR, Marmion VJ (1976). Rapid pneumatic and Mackey-Marg applanation tonometry to evaluate the postural effect on intraocular pressure. Br J Ophthalmol..

[6] Tarkkanen A, Leikola J (1967). Postural variations of the intraocular pressure as measured with the Mackay-Marg tonometer. Acta Ophthalmol (Copenh)..

[7] Prata TS, De Moraes CG, Kanadani FN, Ritch R, Paranhos A Jr (2010). Postureinduced intraocular pressure changes: considerations regarding body position in glaucoma patients. Surv Ophthalmol..

[8] Chiquet C, Custaud MA, Le Traon AP, Millet C, Gharib C, Denis P (2003). Changes in intraocular pressure during prolonged (7-day) head-down tilt bedrest. J Glaucoma..

[9] Hirooka K, Takenaka H, Baba T, Takagishi M, Mizote M, Shiraga F (2009). Effect of trabeculectomy on intraocular pressure fluctuation with postural change in eyes with open-angle glaucoma. J Glaucoma..

[10] Kiuchi T, Motoyama Y, Oshika T (2010). Postural response of intraocular pressure and visual field damage in patients with untreated normal-tension glaucoma. J Glaucoma..

[11] Liu JH, Zhang X, Kripke DF, Weinreb RN (2003). Twenty-four-hour intraocular pressure pattern associated with early glaucomatous changes. Invest Ophthalmol Vis Sci..

[12] Longo A, Geiser MH, Riva CE (2004). Posture changes and subfoveal choroidal blood flow. Invest Ophthalmol Vis Sci..

[13] Parsley J, Powell RG, Keightley SJ, Elkington AR (1987). Postural response of intraocular pressure in chronic open-angle glaucoma following trabeculectomy. Br J Ophthalmol..

[14] Carlson KH, McLaren JW, Topper JE, Brubaker RF (1987). Effect of body position on intraocular pressure and aqueous flow. Invest Ophthalmol Vis Sci..

[15] Weinreb RN, Cook J, Friberg TR (1984). Effect of inverted body position on intraocular pressure. Am J Ophthalmol..

[16] Jain MR, Marmion VJ (1976). Rapid pneumatic and Mackey-Marg applanation tonometry to evaluate the postural effect on intraocular pressure. Br J Ophthalmol..

[17] Sit AJ, Nau CB, McLaren JW, Johnson DH, Hodge D (2008). Circadian variation of aqueous dynamics in young healthy adults. Invest Ophthalmol Vis Sci..

[18] Kontiola AI (2000). A new induction-based impact method for measuring intraocular pressure. Acta Ophthalmol Scand..

[19] Mosaed S, Chamberlain WD, Liu JH, Medeiros FA, Weinreb RN (2008). Association of central corneal thickness and 24-hour intraocular pressure fluctuation. J Glaucoma..

[20] Loewen NA, Liu JH, Weinreb RN (2010). Increased 24-hour variation of human intraocular pressure with short axial length. Invest Ophthalmol Vis Sci..

[21] Liu JH, Kripke DF, Twa MD, Gokhale PA, Jones EI, Park EH, Meehan JE, Weinreb RN (2002). Twenty-four-hour pattern of intraocular pressure in young adults with moderate to severe myopia. Invest Ophthalmol Vis Sci..

[22] Sultan M, Blondeau P (2003). Episcleral venous pressure in younger and older subjects in the sitting and supine positions. J Glaucoma..

[23] Blondeau P, Tetrault JP, Papamarkakis C (2001). Diurnal variation of episcleral venous pressure in healthy patients: a pilot study. J Glaucoma..

[24] Friberg TR, Sanborn G, Weinreb RN (1987). Intraocular and episcleral venous pressure increase during inverted posture. Am J Ophthalmol..

[25] Sit AJ, Weinreb RN, Crowston JG, Kripke DF, Liu JH (2006). Sustained effect of travoprost on diurnal and nocturnal intraocular pressure. Am J Ophthalmol..

[26] Zhen Y, Wang H, Hao J, Ding J, Wang N (2014). Posture-induced intraocular pressure measurements. Zhonghua Yan Ke Za Zhi..

[27] Liu JH, Sit AJ, Weinreb RN (2005). Variation of 24-hour intraocular pressure in healthy individuals: right eye versus left eye. Ophthalmology.

[28] Moster SJ, Fakhraie G, Venketesh R, Moster ML, Zhao Y, Moster MR (2012). Relationship of central corneal thickness to postural IOP changes in patients with and without glaucoma in southern India. Int Ophthalmol..

[29] Barkana Y (2014). Postural change in intraocular pressure: a comparison of measurement with a Goldmann tonometer, Tonopen XL, pneumatonometer,and HA-2. J Glaucoma..

[30] Mosaed S, Liu JH, Weinreb RN (2005). Correlation between office and peak nocturnal intraocular pressures in healthy subjects and glaucoma patients. Am J Ophthalmol..

[31] Mansouri K, Weinreb RN, Liu JH (2012). Effects of aging on 24-hour intraocular pressure measurements in sitting and supine body positions. Invest Ophthalmol Vis Sci..

